# GnRH Antagonist Protocol Versus GnRH Agonist Long Protocol: A Retrospective Cohort Study on Clinical Outcomes and Maternal-Neonatal Safety

**DOI:** 10.3389/fendo.2022.875779

**Published:** 2022-06-29

**Authors:** Jieru Zhu, Weijie Xing, Tao Li, Hui Lin, Jianping Ou

**Affiliations:** Center for Reproductive Medicine, The Third Affiliated Hospital of Sun Yat-sen University, Guangzhou, China

**Keywords:** GnRH antagonist, GnRH agonist, fresh embryo transfer, live birth, obstetric, perinatal, safety

## Abstract

**Objective:**

To evaluate the clinical outcomes and maternal-neonatal safety of gonadotropin releasing hormone antagonist (GnRH-ant) and gonadotropin releasing hormone agonist (GnRH-a) protocols.

**Methods:**

A total of 2505 women undergoing their first *in vitro* fertilization (IVF)/intracytoplasmic sperm injection (ICSI) were retrospectively analyzed. Patients were divided into GnRH-ant group (n = 1514) and GnRH-a group (n = 991) according their stimulation protocol. Propensity Score Matching (PSM) was used for balancing the baseline of two groups. The pregnancy outcomes were analyzed in fresh transfer cycles, and the obstetric and perinatal outcomes were calculated in singleton live births of fresh cycles. The primary outcome was the live birth rate. The secondary outcome measures were maternal complications, preterm birth rate, low birthweight rate, multiple pregnancy rate, and moderate-severe OHSS rate.

**Results:**

After 1:1 PSM, baseline characteristics of the GnRH-ant group and GnRH-a group were matched and assigned 991 cycles in each group. Before PSM, there were 700 fresh cycles including 237 singleton live births in the GnRH-ant group and 588 fresh cycles including 187 singleton live births in the GnRH-a group. After PSM, there were 471 fresh cycles including 166 singleton live births in the GnRH-ant group and 588 fresh cycles including 187 singleton live births in the GnRH-a group. No significant differences were observed in the live birth rate (44.6% vs 48.8%), maternal complications, preterm birth rate (9.0% vs 6.4%), and low birthweight rate (17.5% vs 24.1%) between two groups after PSM (*P* > 0.05). The moderate-severe OHSS rate (2.9% vs 6.0%, *P* = 0.002) and multiple pregnancy rate (24.5% vs 33.1%, *P* = 0.025) was significantly lower in the GnRH-ant group than that in the GnRH-a group after PSM.

**Conclusion:**

GnRH-ant protocol was comparable with GnRH-a protocol in clinical outcomes, obstetric and perinatal outcomes, and with a lower risk of OHSS. For those who want to get an effective and safe outcome, and a shorter treatment period, GnRH-ant is a suitable choice.

## 1 Introduction

Gonadotropin releasing hormone agonist (GnRH-a) has been applied in controlled ovarian stimulation (COS) in assisted reproductive technology (ART) for several decades (1). It binds the GnRH receptor of the pituitary gland and then greatly depletes the receptors, thereby reducing the secretion of follicle stimulating hormone (FSH) and luteinizing hormone (LH). Thanks to the effect of pituitary down- regulation of GnRH-a, the occurrence of premature LH surge is prevented and the homogeneity of follicle development improves (2). However, due to the ‘flare up’ effect and subsequent inhibition of the pituitary gland, the dosage and duration of gonadotropin also increase, and may lead to luteal insufficiency and increase the incidence of ovarian hyperstimulation syndrome (OHSS) (3). Recently, gonadotropin releasing hormone antagonist (GnRH-ant) protocol is increasingly favored in clinical practice because of its physiological advantages. GnRH-ant directly competes with the receptor for binding without pituitary stimulation, therefore inhibiting the secretion of endogenous gonadotropin in a short time. Its strengths include lower dosage and shorter duration of medication, and a reduction of the risk of OHSS (4).

Considerable stimulation outcomes such as the quality of oocytes and embryos, and pregnancy outcomes especially live birth rate are the concerns of reproductive doctors. Several studies have focused on these indicators but the results are controversial (5–7). Evidence shows ART may be related to a higher risk of maternal complications and adverse neonatal outcomes including preterm birth, low birth weight compared with natural pregnancy (3, 8, 9). GnRH-ant protocol and GnRH-a protocol are two mainstream protocols in reproductive centers of China. Few studies have simultaneously analyzed the efficiency and maternal-neonatal safety of the two stimulation protocols, which is essential for reproductive doctors to make the decision for women undergoing *in vitro* fertilization (IVF) or intracytoplasmic sperm injection (ICSI).

The aim of this study was to evaluate the clinical outcomes and maternal-neonatal safety of GnRH-ant protocol and GnRH-a protocol.

## 2 Materials and Methods

### 2.1 Study Design and Population

This is a retrospective cohort study. The information of patients was obtained from the electronic medical records system. Patients receiving their IVF/ICSI treatment in the Center for Reproductive Medicine, The Third Affiliated Hospital of Sun Yat-sen University who met the inclusion criteria and exclusion criteria from January 2016 to May 2021 were retrospectively analyzed. Inclusion criteria (1): Patients undergoing their first IVF/ICSI cycles; (2) Infertility caused by only pelvic oviduct disorder or male sterility; (3) Age between 21~39 years old; (4) Body mass index (BMI) between 18.5~23.9 kg/m^2^; (5) Menstrual cycle between 21~35 days; (6) Received GnRH-ant protocol or GnRH-a protocol for their COS treatment. Patients with uterine malformations, endometriosis, polycystic ovarian syndrome (PCOS), and physical diseases such as hypertension and diabetes mellitus were excluded. Finally, a total of 2505 women were included in the present study, and 1514 women underwent treatment with GnRH-ant protocol and 991 patients underwent treatment with GnRH-a protocol.

### 2.2 Controlled Ovarian Stimulation Protocols

#### 2.2.1 GnRH Antagonist Protocol

Gonadotropin (Merck Serono, Switzerland; MSD, USA; Lizhu Pharmaceutical Trading, China) was administered on the second or third day of the menstrual cycle with a dose of 75~300 IU, depending on the age, BMI, and ovarian reserve of patients. Cetrorelix (Merck Serono, Switzerland) or ganirelix (Organon, The Netherlands; Zhengdatianqing Pharmaceutical Group, China) of 0.25 mg/day was initiated once the diameter of dominant follicle >14 mm or serum estradiol (E_2_) >400 ng/mL or serum luteinizing hormone (LH) >10 IU/L (10), continue to trigger day.

#### 2.2.2 GnRH Agonist Long Protocol

Subcutaneous injection of triptorelin (Ipsen, France; Ferring GmbH, Germany) at a dose of 0.1 mg/day was commenced in the middle luteal phase of the previous menstrual cycle. If the pituitary down-regulation criteria achieved (serum E_2_ <50 pg/mL, FSH <5 IU/L, LH <5 IU/L and the endometrial thickness <5 mm), gonadotropin (Merck Serono, Switzerland; MSD, USA; Lizhu Pharmaceutical Trading, China) was administered of 75~300 IU, based on the age, BMI, and ovarian reserve of patients, until the trigger day.

### 2.3 Trigger, Embryo Transfer, and Luteal Support

6000 IU human chorionic gonadotropin (hCG. Lizhu Pharmaceutical Trading, China) or 250 μg rhCG (Merck Serono, Italy) was injected when at least two follicles >18 mm in diameter. Part of patients with a high risk of OHSS from GnRH-ant cycles was administered 0.2 mg GnRH-a or GnRH-a plus hCG for trigger. Oocyte pick-up (OPU) was performed after 34~36 hours. Fresh embryo transfer was carried out 3 days (cleavage embryo) or 5 days (blastocyst) after OPU. Whole embryos were frozen if patients with a high risk of OHSS, high progesterone level, severe hydrosalpinx, or endometrial polyp. Oral progesterone combined vaginal progesterone or intramuscular progesterone was used for luteal support since the day of OPU for fresh transfer cycles.

### 2.4 Follow-Up

The obstetrical complications and neonatal information were obtained from the medical records if women receiving their prenatal care and delivery in our hospital, otherwise women were contacted by telephone during pregnancy and after delivery.

### 2.5 Outcome Measures

To minimize confounding factors of comparing the two stimulation protocols, all the pregnancy indexes including implantation rate, biochemical pregnancy rate, clinical pregnancy rate, multiple pregnancy rate and live birth rate were analyzed in fresh transfer cycles. The indexes of maternal complications, preterm birth rate, birth weight, low birthweight rate, macrosomia rate and neonatal malformation rate were counted in singleton live births of fresh cycles. The primary outcome was the live birth rate, which was defined as the rate of at least one live-born baby in one fresh transfer cycle. The secondary outcome measures were maternal complications, preterm birth rate, low birthweight rate, multilpe pregnancy rate, and moderate-severe OHSS rate. Preterm birth was defined as live birth greater than 26 and less than 37 weeks’ gestation. Low birthweight was defined as <2500 g birth weight, and macrosomia rate was defined as ≥4000 g birth weight.

### 2.6 Statistical Analysis

Data analysis was performed by IBM SPSS Statistics software, version 26.0. The K-S test was used for the normality test. The Student’s t-test was used to compare the measurement data conforming to normal distribution, and the results were expressed by mean ± standard deviation (SD). Otherwise, the Mann-Whitney U-test was adopted, and the results were expressed by median (quartile range). The Chi-square test was performed for the comparison of counted data, and the results were expressed by percentage (%). Propensity Score Matching (PSM) was used for sampling by 1:1 matching with ‘maximize execution performance’ and caliper (0.02) to balance the baseline of the two groups. The significant level was set at P<0.05.

## 3 Results

### 3.1 Subjects and Stimulation Characteristics

A total of 2505 women were included in this study and divided into the GnRH-ant (n = 1514) and GnRH-a (n = 991) groups. The results of the normality test showed that all the measurement data in the present study were non-normally distributed. Before PSM, significant differences existed in age, basal FSH, basal LH (*P* < 0.05). After 1:1 PSM, patients in the two groups were matched in age, basal FSH, basal LH, and assigned 991 cycles in each group (**Table 1**).

**Table 1:** Baseline information and stimulation characteristics of women before and after PSM.

	Before matched/After matched	GnRH-ant group	GnRH-a group	P value
Cycles/(n)	B	1514	991	
	A	991	991	
Age/(year)	B	31 (28~34)	31 (29~35)	0.002
	A	31 (28~34)	31 (29~35)	0.207
BMI/(kg/m^2^)	B	20.83 (19.72~22.10)	20.81 (19.72~22.10)	0.517
	A	20.83 (19.72~22.04)	20.81 (19.72~22.10)	0.732
Duration of infertility/(year)	B	3.00 (1.75~4.00)	3.00 (1.00~4.00)	0.959
	A	3.00 (2.00~4.00)	3.00 (1.00~4.00)	0.887
bFSH (U/L)	B	6.67 (5.66~7.47)	6.84 (5.92~7.84)	<0.001
	A	6.73 (5.72~7.60)	6.84 (5.92~7.84)	0.032
bLH (U/L)	B	5.42 (3.97~6.56)	4.99 (3.76~6.31)	0.001
	A	5.22 (3.91~6.27)	4.99 (3.76~6.31)	0.240
bE_2_ (pg/mL)	B	40.26 (29.78~48.53)	39.44 (29.86~50.71)	0.763
	A	40.14 (29.53~48.02)	39.44 (29.86~50.71)	0.555
Type of infertility				
Primary	B	720/1514 (47.6)	465/991 (46.9)	0.756
	A	467/991 (47.1)	465/991 (46.9)	0.928
Secondary	B	794/1514 (52.4)	526/991 (53.1)	
	A	524/991 (52.9)	526/991 (53.1)	
Fertilization type				
IVF	B	1256/1514 (83.0)	795/991 (80.2)	0.082
	A	821/991 (82.8)	795/991 (80.2)	0.132
ICSI	B	258/1514 (17.0)	196/991 (19.8)	
	A	170/991 (17.2)	196/991 (19.8)	
Duration of stimulation/(days)	B	8 (7~9)	10 (8~11)	<0.001
	A	8 (7~9)	10 (8~11)	<0.001
Dosage of gonadotropins/(IU)	B	1500 (1125~1800)	1950 (1250~2400)	<0.001
	A	1550 (1200~1900)	1950 (1250~2400)	<0.001
E_2_ values on the trigger day/(pg/mL)	B	2682 (1744~3765)	2821 (1920~3880)	0.027
	A	2635 (1701~3699)	2821 (1920~3880)	0.005
No. of oocytes retrieved/(n)	B	12 (7~16)	12 (8~16)	0.290
	A	11 (7~17)	12 (8~16)	0.049
MII rate	B	15596/19019 (82.0)	9921/12611 (78.7)	<0.001
	A	9992/12171 (82.1)	9921/12611 (78.7)	<0.001
Fertilization rate	B	14822/19019 (77.9)	9679/12611 (76.8)	0.014
	A	9492/12171 (78.0)	9679/12611 (76.8)	0.020
D3 high qualified embryo rate	B	6326/12360 (51.2)	3839/8304 (46.2)	<0.001
	A	4059/7985 (50.8)	3839/8304 (46.2)	<0.001
Moderate-severe OHSS rate	B	44/1514 (2.9)	59/991 (6.0)	0.002
	A	33/991 (3.3)	59/991 (6.0)	0.030

Data are presented as the M(P25~P75) for continuous variables and n (%) for categorical variables. BMI, body mass index; bFSH, basal FSH; bLH, basal LH; bE_2_, basal E_2_; IVF, in vitro fertilization; ICSI, intracytoplasmic sperm injection.

The age, BMI, duration of infertility, basal LH, basal E2, type of infertility, and fertilization type were comparable in the two groups after PSM. The basal FSH level was a bit higher in the GnRH-a group (*P* < 0.05). The duration of stimulation, dosage of gonadotropins, E2 values on the trigger day, No. of oocytes retrieved, moderate-severe OHSS rate were significantly lower in the GnRH-ant group than those in the GnRH-a group (*P* < 0.05). The MII rate, fertilization rate, and D3 high qualified embryo rate were significantly higher in the GnRH-ant group than those in the GnRH-a group (*P* < 0.05) (**Table 1**).

### 3.2 Pregnancy Outcomes of Fresh Transfer Cycles

There were 700 fresh cycles in the GnRH-ant group and 588 fresh cycles in the GnRH-a group before PSM, and 471 fresh cycles in the GnRH-ant group and 588 fresh cycles in the GnRH-a group after PSM. The average number of embryos transferred and the multiple pregnancy rate after fresh embryo transfer was significantly lower in the GnRH-ant group than that in the GnRH-a group no matter before or after PSM (*P* < 0.05). No significant differences were observed in the embryo type of transfer, implantation rate, biochemical pregnancy rate, clinical pregnancy rate, miscarriage rate, and live birth rate of fresh cycles between two groups before and after PSM (*P* > 0.05) (**Table 2**).

**Table 2:** Pregnancy outcomes of fresh cycles before and after PSM matching.

	Before matched/After matched	GnRH-ant group	GnRH-a group	P value
Fresh transferred cycles/(n)	B	700	588	
	A	471	588	
Cleavage embryo or blastocyst transfer				
Cleavage embryo	B	511/700 (73.0)	451/588 (76.7)	0.229
	A	351/471 (74.5)	451/588 (76.7)	0.411
Blastocyst	B	189/700 (27.0)	137/588 (23.3)	
	A	120/471 (25.5)	137/588 (23.3)	
Implantation rate	B	460/1126 (40.9)	436/1030 (42.3)	0.487
	A	301/751 (40.1)	436/1030 (42.3)	0.341
Average No. of embryos transferred	B	2 (1~2, 1.61 ± 0.50)	2 (1~2, 1.75 ± 0.47)	<0.001
	A	2 (1~2, 1.59 ± 0.50)	2 (1~2, 1.75 ± 0.47)	<0.001
Biochemical pregnancy rate	B	29/700 (4.1)	22/588 (3.7)	0.713
	A	23/471 (4.9)	22/588 (3.7)	0.360
Clinical pregnancy rate	B	366/700 (52.3)	329/588 (56.0)	0.188
	A	241/471 (51.2)	329/588 (56.0)	0.121
Multiple pregnancy rate	B	94/366 (25.7)	109/329 (33.1)	0.031
	A	59/241 (24.5)	109/329 (33.1)	0.025
Miscarriage rate	B	23/366 (6.3)	32/329 (9.7)	0.093
	A	14/241 (5.8)	32/329 (9.7)	0.090
Live birth rate	B	329/700 (47.0)	287/588 (48.8)	0.517
	A	210/471 (44.6)	287/588 (48.8)	0.171

Data are presented as the M(P25~P75, mean ± SD) for the average No. of embryos transferred, and the n (%) for categorical variables.

### 3.3 Maternal Complications and Neonatal Outcomes of Singleton Live Births

There were 237 singleton live births of fresh cycles in the GnRH-ant group and 187 singleton live births of fresh cycles in the GnRH-a group before PSM, and 166 singleton live births of fresh cycles in the GnRH-ant group and 187 singleton live births of fresh cycles in the GnRH-a group after PSM. There were no significant differences in the maternal complications such as gestational diabetes mellitus (GDM), gestational hypertension, with GDM and gestational hypertension at the same time, and other complications like anemia, thrombocytopenia, intrahepatic cholestasis of pregnancy (ICP) between the two groups before and after PSM (*P* > 0.05). The preterm birth rate, birth weight, low birthweight rate, macrosomia rate and neonatal malformation rate were comparable between the two groups before and after PSM (*P* > 0.05) (**Table 3**). There was one case of ventricular septal defect (VSD), one case of patent ductus arteriosus (PDA) and one case of persistent left superior vena cava (PLSVC) in the GnRH-ant group, and one case of inherited metabolic disorder (IMD), one case of patent ductus arteriosus (PDA) and one case of patent foramen ovale (PFO) in the GnRH-a group after PSM.

**Table 3:** Obstetric and perinatal outcomes of singleton live births before and after PSM matching.

	Before matched/After matched	GnRH-ant group	GnRH-a group	P value
Singletons live births/(n)	B	237	187	
	A	166	187	
Maternal complications				
No complications	B	190/237 (80.2)	153/187 (81.8)	0.915
	A	132/166 (79.5)	153/187 (81.8)	0.759
GDM	B	28/237 (11.8)	17/187 (9.1)	
	A	22/166 (13.3)	17/187 (9.1)	
Gestational hypertension	B	9/237 (3.8)	8/187 (4.3)	
	A	6/166 (3.6)	8/187 (4.3)	
Both GDM and gestational hypertension	B	5/237 (2.1)	4/187 (2.1)	
	A	3/166 (1.8)	4/187 (2.1)	
Others	B	5/237 (2.1)	5/187 (2.7)	
	A	3/166 (1.8)	5/187 (2.7)	
Preterm birth rate	B	21/237 (8.9)	12/187 (6.4)	0.351
	A	15/166 (9.0)	12/187 (6.4)	0.355
Birth weight	B	3020 (2090~3050)	3020 (2095~3050)	0.613
	A	3025 (2095~3055)	3020 (2095~3050)	0.564
Low birthweight rate	B	41/237 (17.3)	45/187 (24.1)	0.085
	A	29/166 (17.5)	45/187 (24.1)	0.129
Macrosomia rate	B	2/237 (0.8)	6/187 (3.2)	0.076
	A	2/166 (1.2)	6/187 (3.2)	0.207
Neonatal malformation rate	B	3/237 (1.3)	3/187 (1.6)	0.770
	A	3/166 (1.8)	3/187 (1.6)	0.883

Data are presented as the M(P25~P75) for continuous variables and n (%) for categorical variables. GDM, gestational diabetes mellitus.

## 4 Discussion

In recent years, the advantages of GnRH-ant have gradually emerged with its promotion and application in ART treatments. Compared with GnRH-a, GnRH-ant competitively binds to the GnRH receptor of the pituitary gland, without waiting for receptor exhaustion and desensitization, and there is no ‘flare up’ effect. It inhibits the secretion of gonadotropin within a few hours and avoids excessive pituitary inhibition. Therefore, GnRH-ant can effectively reduce the consumption of gonadotropin and greatly shorten the treatment time (11, 12). The results of the present study suggested that the duration of stimulation and the dosage of gonadotropins of GnRH-ant group were significantly lower than those of GnRH-a group, which was consistent with the previous studies (6, 13).

Live birth is the common goal of patients and reproductive doctors. Optimistic ovarian stimulation outcomes are closely related to favourable live birth rate. Although the number of oocytes retrieved of the GnRH-ant group was slightly inferior to the GnRH-a group in our study, the MII rate, fertilization rate, and D3 high qualified embryo rate were significantly higher. These results indicated that the GnRH-ant protocol had a positive effect on follicular maturity, fertilization ability, and embryonic developmental potential (5). In order to obtain more objective results, evaluate the impact of stimulation protocols on subsequent pregnancy outcomes, and avoid some confounding factors of frozen cycles, we only selected fresh cycles to analyze the pregnancy outcomes of the two protocols. The implantation rate, clinical pregnancy rate, and live birth rate were comparable in the GnRH-ant and GnRH-a protocols, suggesting GnRH-ant protocol was as effective as GnRH-a protocol. Recently, a real-world study of 18853 women from China concluded that the cumulative live birth rate (CLBR) was similar in GnRH-ant and GnRH-a groups (5). Its large sample and real-world data provided a powerful reference for clinical practice. Although we focused on live birth rate but not CLBR in the present study, the conclusion was consistent, and was significative for explaining to patients the success chance of one fresh cycle. Several studies have reported live birth rates with GnRH-ant protocol and yielded the same conclusions as to the present study (7, 14, 15). A meta-analysis including 29 randomized controlled trials (RCTs) showed that there were no significant differences in the clinical pregnancy rate, ongoing pregnancy rate, and live birth rate between the GnRH-ant and GnRH-a groups (14). A clinical research reported that there were no significant differences in the clinical pregnancy rate and live birth rate among the modified agonist, mild-stimulation and antagonist protocols (7). Some studies have come to different conclusions. Another meta-analysis suggested that the live birth rate with GnRH-ant protocol averaged 1.5% lower than GnRH-a protocol (16), but it only included 9 RCTs involving 1515 women, the sample of which was too small.

The safety of treatments was another considerable thing, including maternal and neonatal safety. In the present study, the moderate-severe OHSS incident rate and multiple pregnancy rate of GnRH-ant group were significantly lower than those of GnRH-a group, which was consistent with previous reports (17–19). The follicle development of GnRH-ant protocol is not as synchronous as that of the GnRH-a protocol, and the gonadotropin dosage and estrogen levels of trigger day were lower, which may be the reasons for reducing the occurrence of OHSS (17). Because of a bit less number of embryos transferred and slightly lower implantation rate in the GnRH-ant group, its multiple pregnancy rate was lower. Previous studies indicated higher rates of pregnancy complications and adverse neonatal outcomes in women receiving ART (8, 20, 21). All women of fresh transfer cycles will receive exogenous progesterone as luteal support, which increases the risk of insulin resistance and leads to gestational diabetes mellitus (GDM) (22). Studies also suggested that hyperphysiological doses of estrogen were associated with preeclampsia and low birth weight (3, 9, 23). However, few researches focused on the relationship between stimulation protocols and perinatal outcomes. Our results suggested that there were no significant differences in the maternal complications, preterm birth rates, birth weight, low birthweight rates, macrosomia rates, and neonatal malformation rates between GnRH-ant and GnRH-a groups. That was to say, the differences in steroid hormone levels, placental gene imprinting and epigenetic changes caused by the two protocols did not seem to affect perinatal outcomes (21). The results of an RCT including 521 gestations showed that in singletons after fresh embryo transfer, the preterm birth rates, mean birthweight, and low birthweight rates were similar of GnRH-ant and GnRH-a protocols (3). A large prospective, pregnancy and infant follow-up trial indicated that there were more multiple pregnancies in the GnRH agonist, but no significant differences in major congenital malformations in fetuses of GnRH antagonist and GnRH agonist (19). These results were consistent with the results of the present study.

The greatest advantage of this study was using PSM for balancing the baseline characteristics of two groups. Not only the stimulation and pregnancy outcomes, but also the obstetric and perinatal conditions of the two protocols were evaluated with strict inclusion and exclusion criteria. The limitation was that this was a retrospective analysis with a limited sample. The control for confounding factors and the preciseness of the conclusions were not as good as prospective RCTs. The information may be inaccurate because part of women were followed up only by telephone. However, this was a study that was from the real world, which was closer to the clinical practice.

In summary, GnRH-ant protocol was comparable with GnRH-a protocol in clinical outcomes, obstetric and perinatal outcomes, and with a lower risk of OHSS. For patients who want to get an effective and safe outcome, and a shorter treatment period, GnRH-ant is a suitable choice. The results of the present study require a well-designed RCT and larger samples to be identified.

## Data Availability Statement

The original contributions presented in the study are included in the article. Further inquiries can be directed to the corresponding author.

## Ethics Statement

The studies involving human participants were reviewed and approved by the medical ethics committee of Third Affiliated Hospital of Sun Yat-sen University. The patients/participants provided their written informed consent to participate in this study.

## Author Contributions

JZ and JO designed the study. WX selected the study population that met the criteria and exclusion criteria. JZ and WX performed the statistical analysis. JO, TL and HL reviewed the data. JZ drafted the manuscript. All authors contributed to the article and approved the submitted version.

## Conflict of Interest

The authors declare that the research was conducted in the absence of any commercial or financial relationships that could be construed as a potential conflict of interest.

## Publisher’s Note

All claims expressed in this article are solely those of the authors and do not necessarily represent those of their affiliated organizations, or those of the publisher, the editors and the reviewers. Any product that may be evaluated in this article, or claim that may be made by its manufacturer, is not guaranteed or endorsed by the publisher.
